# N6-Methyladenosine Modification Opens a New Chapter in Circular RNA Biology

**DOI:** 10.3389/fcell.2021.709299

**Published:** 2021-07-23

**Authors:** Jun Wu, Xin Guo, Yi Wen, Shangqing Huang, Xiaohui Yuan, Lijun Tang, Hongyu Sun

**Affiliations:** ^1^Department of General Surgery and Pancreatic Injury and Repair Key Laboratory of Sichuan Province, The General Hospital of Western Theater Command, Chengdu, China; ^2^College of Medicine, Southwest Jiaotong University, Chengdu, China; ^3^Laboratory of Basic Medicine, The General Hospital of Western Theater Command, Chengdu, China

**Keywords:** N6-methyladenosine, circular RNAs, metabolism, immunity, diseases

## Abstract

As the most abundant internal modification in eukaryotic cells, N6-methyladenosine (m6A) in mRNA has shown widespread regulatory roles in a variety of physiological processes and disease progressions. Circular RNAs (circRNAs) are a class of covalently closed circular RNA molecules and play an essential role in the pathogenesis of various diseases. Recently, accumulating evidence has shown that m6A modification is widely existed in circRNAs and found its key biological functions in regulating circRNA metabolism, including biogenesis, translation, degradation and cellular localization. Through regulating circRNAs, studies have shown the important roles of m6A modification in circRNAs during immunity and multiple diseases, which represents a new layer of control in physiological processes and disease progressions. In this review, we focused on the roles played by m6A in circRNA metabolism, summarized the regulatory mechanisms of m6A-modified circRNAs in immunity and diseases, and discussed the current challenges to study m6A modification in circRNAs and the possible future directions, providing a comprehensive insight into understanding m6A modification of circRNAs in RNA epigenetics.

## Introduction

Epigenetic modifications in RNA have been found to playimportant regulatory roles in a variety of physiological processes and disease progressions. To date, over 160 types of chemical modifications have been identified in RNA molecules, such as N6-methyladenosine (m6A), 5-methylcytosine (m5C), N1-methyladenosine (m1A), 5-hydroxymethylcytosine (5hmC), N6, 2′-Odimethyladenosine (m6Am), 7-methylguanine (m7G), and so on (Roundtree et al., 2017a; Frye et al., 2018). Among them, m6A has been considered as the most abundant internal modification in RNA, which was discovered from methylated nucleosides in mRNA of Novikoff hepatoma cells in the early 1970s (Desrosiers et al., 1974; Roundtree et al., 2017a). With the development of next-generation sequencing and bioinformatics, a large number of m6A modifications in the transcriptome were discovered and annotated, including mRNAs and non-coding RNAs (Zhou et al., 2017; Frye et al., 2018; He et al., 2020; Sekar and Lakshmanan, 2020; Zhu et al., 2020). In mRNA, the m6A modification has been extensively studied and reviewed by several excellent reviews (Roundtree et al., 2017a; Zaccara et al., 2019; Zhu et al., 2020; Jiang et al., 2021).

Up to now, accumulating evidence has demonstrated that circular RNAs (circRNAs) play important roles in the occurrence, development and prognosis of various diseases, such as cardiovascular diseases (Aufiero et al., 2019; Zhang and Huang, 2020), immune diseases (Chen X. et al., 2019), tumors (Meng S. et al., 2017), skin diseases (Wu et al., 2020), and so on. However, the regulatory role of m6A in circRNAs remains unclear. It was not until 2017 that circRNAs were found to be modified by m6A (Zhou et al., 2017). Based on the important roles of m6A in RNA epigenetic modifications, predictably and rapidly, a large number of recent studies have demonstrated that m6A acts as a key regulator to affect circRNAs functions, thereby participating in development and disease progressions. Therefore, in this review, we focused on the roles of m6A modification in circRNAs during circRNA metabolism and discussed their biological consequences in human development and diseases based on current studies. Importantly, this review will provide a comprehensive understanding of m6A in circRNA biology.

## Biogenesis, Characteristics and Functions of circRNAS

circRNAs are a class of covalently closed circular RNA molecules thatlack 5′ caps and 3′ tails, which were discovered in the 1970s (Hsu and Coca-Prados, 1979; Chen and Yang, 2015). In a long time afterward, they were thought to be byproducts of splicing (Sanger et al., 1976; Panda et al., 2017). In recent years, a large number of circRNAs have been discovered and annotated with the development of next-generation sequencing technologies and bioinformatic approaches (Xia et al., 2017). The circRNAs are mainly generated by the back-splicing of pre-mRNAs. Currently, three mechanisms were proposed regarding the biogenesis of circRNAs, namely lariat-driven circularization (Zaphiropoulos, 1996; Jeck et al., 2013), intron pairing-driven circularization (Jeck et al., 2013; Zhang et al., 2014; Ivanov et al., 2015) and RBP-driven circularization (Conn et al., 2015; Kramer et al., 2015). In addition, circRNAs are also generated by pre-transfer ribonucleic acid (tRNA), and pre-ribosomal ribonucleic acid (rRNA) molecules (Zhang et al., 2017). According to their origin, the formed circRNAs can be classified into four categories (Zhang et al., 2017): intronic circRNAs (CiRNAs), exon-intron circRNAs (EIciRNAs), exonic circRNAs (EcRNAs), and others (such as the circRNAs that are derived from tRNA and rRNA).

circRNAs were expressed widely in tissue, cell, and developmentalstage-specific manners (Memczak et al., 2013; Nicolet et al., 2018) and existedin most mammals (Memczak et al., 2013), plants (Wang et al., 2014), and virus (Nahand et al., 2020). For example, in human heart, about 9% of the expressed genes can produce circRNAs (Aufiero et al., 2018), while in brain, 20% of the genes can produce circRNAs (Rybak-Wolf et al., 2015). In addition, different circular junctions can be generated from the same gene (Nicolet et al., 2018). These discoveries indicated the important regulatory roles of circRNAs. Another characteristic is that circRNAs are more stable than the linear RNAs and have long half-lives than 48 h (Jeck et al., 2013). Due to the covalently closed circular structure without 5′ caps and 3′ tails, circRNAs can resist the foreign chemicals or exonucleases (Jeck et al., 2013; Aufiero et al., 2019). Therefore, despite the low expression level, circRNAs can be accumulated to the relatively high levels.

A large number of studies have revealed the functions of circRNAs. (1) microRNA (miRNA) sponges. Cytoplasmic circRNAs can bind specific miRNAs by miRNA response elements to prevent the interplays with target mRNAs (Thomson and Dinger, 2016; Cheng et al., 2019). (2) Interaction with proteins. Some circRNAs that contain protein binding sites have the protein binding abilities to regulate their activity and localization (Du W. W. et al., 2016; Barbagallo et al., 2019). (3) Templates for protein synthesis. Due to the lack of 5′-end, the translation of circRNAs only is initiated by the cap-independent mechanisms. This function of circRNAs will be discussed in detail in following section. (4) Regulation of gene transcription. Some circRNAs located in nucleus, such as circ-EIF3J and circ-PAIP2, can combine with the U1 snRNP to further interact with RNA Pol II and enhance the expression of their parental genes in HeLa and HEK293 cells (Li et al., 2015).

## m6A Modification of circRNA

### Widely Existed m6A Modification of circRNAs

Through depletion of rRNA, treatment with RNase R to digest linear RNAs and m6A-modified RNA immunoprecipitation sequencing (MeRIP-seq), m6A modification of circRNAs has been found to exist widely. In plant, Wang et al. (2020) found that about 10% of the EcRNAs contained m6A sites in moso bamboo. In human embryonic stem cells (hESCs) and HeLa cells, Zhou et al. (2017) identified 1,404 m6A circRNAs and 987 m6A circRNAs, respectively, and they found that m6A circRNAs were expressed in cell-type-specific methylation patterns. In a rat model of hypoxia mediated pulmonary hypertension (HPH), Su et al. (2020) identified 1130 m6A circRNAs in total. In the lens epithelium cells from age-related cataract (ARC), Li et al. (2020) identified 4876 m6A peaks within circRNAs. Besides, in HPH, 80% of m6A circRNAs were derived from protein-coding genes, while in ARC, over 70% of circRNAs were derived from sense overlapping. These studies suggested that m6A modification of circRNAs was widely existed and might play important roles in diseases. In addition, the m6A sites in circRNAs may be distinct from the corresponding mRNAs. In hESCs cells, Zhou et al. (2017) reported that 33% of m6A circRNAs were produced from genes that encode m6A mRNAs methylated on different exons, while 26% of m6A circRNAs were produced from genes that encode mRNAs without detectable m6A modification (Zhou et al., 2017). Another phenomenon is that m6A sites in mRNAs are most common in the last exon (Meyer et al., 2012), while circularization of the last exon of genes is uncommon (Zhang et al., 2014). These evidence suggested that circRNAs exhibit the patterns of m6A modification that are distinct from those of mRNAs.

### m6A Regulators of circRNA

As the most abundant mRNA internal modification, the functions of m6A on mRNA are mediated through three homologous factors (Zaccara et al., 2019), namely so called “writers” (methyltransferase), “erasers” (demethylase), and “readers” (recognition). In circRNAs, m6A modification was found to be written, read and erased by the same regulators with mRNA (Zhou et al., 2017).

#### m6A Writers

The m6A is installed by a multi-subunit complex (Zaccara et al., 2019). This complex consists of two core components (methyltransferase-like 3 protein (METTL3) and METTL14) and other accessory regulatory subunits [Wilm’s tumor-1-associated protein (WTAP), VIRMA (Virilizer), E3 ubiquitin-protein ligase HAKAI, RNA recognition motif 15/15B (RBM15/15B), Zinc finger CCCH domain-containing protein 13 (ZC3H13)] (Zaccara et al., 2019; Gu et al., 2021; Jiang et al., 2021). METTL3 is the catalytic core of the multi-subunit complex (Bokar et al., 1997; Liu et al., 2014). METTL14 contains the RNA-binding site and is an allosteric activator of the enzymatic activity of METTL3 (Liu et al., 2014; Zaccara et al., 2019). WTAP is essential for m6A formation and it is responsible for localizing METTL3–METTL14 heterodimers to transcription sites (Ping et al., 2014). VIRMA may modulate region-selective methylation of sites in 3′ UTR and location near stop codons (Yue et al., 2018). RBM15/15B are the mediators of methylation specificity, which facilitate the recruitment of the writer complex to specific sites (Patil et al., 2016; Zaccara et al., 2019). ZC3H13 is a WTAP interactor, which acts as a bridge between RBM15 and WTAP (Knuckles et al., 2018). HAKAI functions as an E3 ubiquitin ligase, which was first identified in the WTAP interaction proteome (Horiuchi et al., 2013). Deletion of HAKAI results in a partial reduction in global m6A levels (Růžička et al., 2017). Notably, METTL16 is the new identified methyltransferase that can regulate S-adenosylmethionine homeostasis and can methylate long non-coding RNA and U6 small nuclear RNA (Brown et al., 2016; Pendleton et al., 2017; Warda et al., 2017). Currently, only few m6A residues in poly(A) RNA were found to be catalyzed by METTL16 (Brown et al., 2016; Pendleton et al., 2017; Warda et al., 2017).

#### m6A Erasers

m6A is a reversible process, which can be removed by demethylases. There are two demethylases, namely obesity-associated protein (FTO) and AlkB homolog 5 (ALKBH5). Both of them belong to the α-ketoglutarate-dependent dioxygenase AlkB-like superfamily and they remove m6A through Fe^2+^ and α-ketoglutarate-dependent manner (Jia et al., 2011; Zheng et al., 2013). Under physiological conditions, the demethylases appear to have a limited role, indicating that they function in specific tissues, or under specific stress and disease-relevant conditions (Zhao et al., 2017).

#### m6A Readers

m6A readers are a class of RNA binding proteins, which can recognize and bind to m6A sites, thereby resulting in different downstream effects, such as splicing, export and translation of m6A RNAs (Xiao et al., 2016; Roundtree et al., 2017b; Yang et al., 2017). In YT521-B homology (YTH) domain family, there are five m6A readers identified, namely YTHDC1, YTHDC2, YTHDF1, YTHDF2, and YTHDF3 (Liu et al., 2015). Among them, YTHDF1, YTHDF2, and YTHDF3 are mainly located in the cytoplasm. YTHDC1 is mainly found in the nucleus and YTHDC2 is located in both cytoplasm and nucleus. YTHDC1 affects their splicing (Kasowitz et al., 2018) and export (Roundtree et al., 2017b); YTHDC2 affects their degradation and translation (Hsu et al., 2017); YTHDF1 promotes translation (Wang et al., 2015); YTHDF2 promotes degradation (Du H. et al., 2016) and YTHDF3 promotes the translation and degradation (Shi et al., 2017). The other m6A readers include insulin-like growth factor 2 mRNA binding proteins 1-3 (IGF2BP1-3), heterogeneous nuclear ribonucleoprotein (HNRNP) family (HNRNPC, HNRNPA2B1, and HNRNPG), fragile X mental retardation protein (FMRP) and eukaryotic initiation factor 3 (eIF3). IGF2BPs can regulate gene expression by enhancing the stability of target RNAs (Huang H. et al., 2018). FMRP can promote nuclear export (Edens et al., 2019). eIF3 can facilitate cap-independent translation (Meyer et al., 2015).

## Roles of m6A in circRNA Metabolism

**FIGURE 1:**
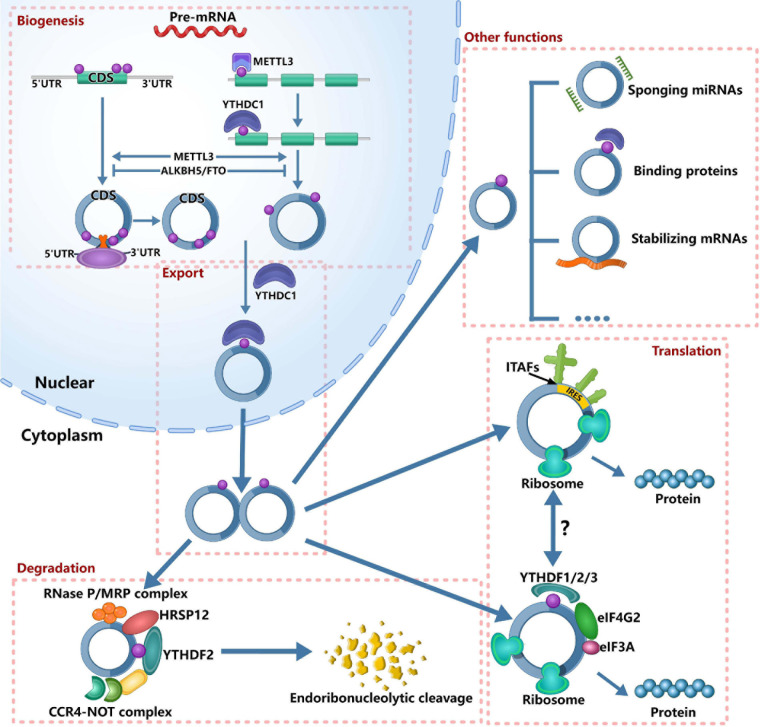
Roles of m6A in circRNA metabolism. It’s has confirmed that m6A can regulate circRNA metabolism, including circRNAs biogenesis, translation, degradation and cellular localization. On the one hand, m6A sites located around the start and stop codons in linear mRNAs can recruit spliceosome, leading to back splicing and circRNA production. On the other hand, m6A deposition and YTHDC1 binding to exons can regulate circularization. In the nucleus, the m6A can bind specific nuclear reader proteins, mainly YTHDC1, which can promote the export of circRNAs. Upon circRNAs export to the cytoplasm, m6A binds to specific reader proteins and other proteins to stabilize some mRNAs. The nuclear export of circRNAs also affects its miRNA sponges. The translation of circRNAs is only in cap-independent translation initiation mechanisms: IRES-dependent initiation of translation and m6A-dependent initiation of translation. m6A-driven translation of circRNA requires eIF4G2 and YTHDF3 and is enhanced by METTL3/14, inhibited by FTO. IRES-driven translation and m6A-driven translation may have interplays. Finally, m6A-modified circRNAs are endoribonuclease-cleaved via the YTHDF2-HRSP12-RNase P/MRP axis.

Currently, several studies have found that m6A modification can affect the “fate” of m6A-modified circRNAs (Figure 1), including circRNAs biogenesis, translation, degradation and cellular localization, indicating that m6A plays important roles in circRNA metabolism. Through participating in circRNA metabolism, m6A can regulate many physiological and pathological processes. For example, during the nuclear phase, m6A can be read by specific nuclear readers and promote the nuclear export of circRNAs. Upon exporting to the cytoplasm, m6A can be read by specific cytosolic reader proteins, thereby affecting the function of circRNAs. Therefore, this section aimed to discuss the roles of m6A in circRNA metabolism comprehensively.

### m6A and Biogenesis of circRNA

circRNA is conserved evolutionarily and is expressed in cell, tissue and developmental stage-specific expression patterns (Memczak et al., 2013; Hsiao et al., 2017; Nicolet et al., 2018), suggesting that circRNAs play an important regulatory role in different physiological and pathological processes. Therefore, it is essential to understand the regulation regarding the biogenesis of circRNAs. The biogenesis of circRNA is distinct from canonical splicing, and it is generated through a back-splicing orchestrated by the spliceosome machinery (Aufiero et al., 2019). Recently, Tang et al. (2020) reported that m6A could promote the biogenesis of circRNA in male germ cells. They found that, for open reading frames (ORFs)-containing circRNAs during murine spermatogenesis, the back splicing occurred mostly at m6A enriched sites, while these m6A sites were usually located around the start and stop codons in linear mRNAs. To further establish the cause–effect relationship between m6A and circRNAs, Tang et al. (2020) knocked out ALKBH5 and METTL3, respectively. After knocking out ALKBH5 in spermatogenic cells, the m6A level was significantly increased compared with wild-type controls. Consistently, the circRNAs abundance was increased in Alkbh5-null spermatogenic cells. After knocking out METTL3, they identified much fewer circRNAs. Similarly, Di Timoteo et al. (2020) also reported that the level of circRNA was decreased in HeLa cells after knocking out METTL3. Interestingly, they found the consistent decrease of circ-ZNF609 after knocking out YTHDC1 in HeLa, RD, RH4, and HEK293T cells. Further analysis found that m6A deposition and YTHDC1 binding to exons that undergo circularization were significantly correlative, and circRNAs containing total-only m6As were more represented among those decreasing upon depletion of both YTHDC1 and METTL3 compared with those affected by either METTL3 or YTHDC1 knockdown alone. These evidence indicated that m6A regulated the biogenesis of circRNAs in an METTL3/YTHDC1-dependent manner. In colorectal cancer, METTL3 could increase the expression of circ1662 (Chen et al., 2021). Therefore, based on the above evidence, we can confirm that m6A modification can increase the biogenesis of circRNAs. However, more detailed information is needed to understand how m6A regulates the biogenesis of circRNAs.

### m6A and Translation of circRNA

Although circRNA is considered as non-coding RNA for a long time, accumulating evidence has shown that some circRNAs haveprotein-coding potential and can be translated (Legnini et al., 2017; Pamudurti et al., 2017; Yang et al., 2017, 2018; Zhang et al., 2018a, b; Liang et al., 2019). More recently, Huang et al. (2021) reported a database, TransCirc,^1^ to help investigate the circRNAs that have translation capacity. However, the mechanisms about the translation of circRNAs remain largely unknown. As we all know, the translation initiation of mRNA is dependent on the cap structure of 5′-end, which contains a 7-methyl guanosine (m7G) that can be recognized by the eukaryotic translation initiation factor 4E (eIF4E) (Gross et al., 2003). However, circRNA is a covalently closed RNA molecule without 5′ caps and 3′ tails (Meng X. et al., 2017), so that circRNA is translated in cap-independent translation initiation mechanisms. Currently, two mechanisms are reported in translation initiation of circRNA: internal ribosome entry site (IRES)-dependent initiation of translation and m6A-dependent initiation of translation. IRESs are sequences that form secondary structures on RNA and can initiate translation through recruiting ribosomes by IRES-transacting factors (ITAFs) in the absence of canonical translation initiation factors (Godet et al., 2019). At first, the fact that circRNAs can be translated is proved by IRES-driven pathway (Chen and Sarnow, 1995). Afterward, more and more circRNAs that can be translated by IRES-driven pathway were reported, such as circZNF609 (Legnini et al., 2017), circMbl (Pamudurti et al., 2017), circSHPRH (Zhang et al., 2018a), circFBXW7 (Yang et al., 2018), circLINC-PINT (Zhang et al., 2018b), circb-catenin (Liang et al., 2019).

Interestingly, the discovery of m6A that can initiate the translation of circRNA expands the coding landscape of human transcriptome (Yang et al., 2017). Yang et al. (2017) found that the m6A-driven translation of circRNAs was widespread, with hundreds of endogenous circRNAs having translation potential, by the analysis of polysome profiling, computational prediction and mass spectrometry. Mechanistically, they found that the m6A-driven translation of circRNA required initiation factor eIF4G2 and m6A reader YTHDF3, and was enhanced by methyltransferase METTL3/14, inhibited by demethylase FTO (Yang et al., 2017). When inserting a short fragment (19nt) containing different copies of consensus m6A motifs (RRACH) before the start codon of circRNA reporter in 293 cells, the protein could be detected. Moreover, single m6A site has similar translation efficiency compared to circRNA with two m6A sites (Yang et al., 2017). These evidence suggested that m6A motif was essential for initiating translation. In human papillomavirus (HPV), m6A-modified circE7 could be translated to produce E7 oncoprotein (Zhao et al., 2019).

In addition to initiating the translation of circRNAs, m6A may also regulate the translation of circRNAs (Yang et al., 2017). For example, circZNF609 could be translated by IRES-driven pathway (Legnini et al., 2017). A recent study reported that two mutant at m6A sites displayed approximately 50% reduction of circ-ZNF609 translation (Di Timoteo et al., 2020). Further analysis found that m6A regulated its translation through recognition by YTHDF3 and eIF4G2 (Di Timoteo et al., 2020). This study suggested that the two cap-independent translation of circRNA might have interplays. However, the relationships between the two cap-independent translations need further investigations.

In mRNAs, studies have shown that m6A regulates their translation under stress response (Zhou et al., 2015, 2018). Yang et al. (2017) found that m6A-mediated circRNA translation was increased under the heat shock condition and its mechanism might be the translocation of YTHDF2 from cytosol into nucleus upon heat shock to block the m6A “eraser” FTO (Zhou et al., 2015; Yang et al., 2017). These evidence suggested that m6A-mediated circRNA translation played important roles in cellular stress response. In the future, to broaden our understanding regarding the significance of circRNA translation in the specific conditions, it is essential to explore the conditions that affect the m6A-mediated circRNA translation.

### m6A and Degradation of circRNA

Due to the covalently closed circular structure without 5′ caps and3′ tails, circRNAs can resist the foreign chemicals or exonucleases (Jeck et al., 2013; Aufiero et al., 2019). Therefore, circRNAs are more stable than the linear RNAs and are not degraded readily by RNase R, resulting in their long half-lives exceeding 48 h (Jeck et al., 2013; Suzuki and Tsukahara, 2014). Thus, there are few studies that reported the degradation of circRNA.

Hansen et al. (2011) reported that circRNAs with near perfect complementary miRNA target sites could be degraded in an Ago2-slicer-dependent manner. Ago2 is a member of the Argonaute protein family, which is important for RNA interference (Suzuki and Tsukahara, 2014) and can mediate the degradation of circRS-7 (Hansen et al., 2011). However, only a few circRNAs have miRNA sponge function or specific miRNA target sites. Jia et al. (2019) found that the depletion of GW182 (a key component of the P-body and RNA interference machine) could lead to the accumulation of endogenous circular transcripts. However, the depletion of other P-body components or RNAi complex factors did not have similar effects. So, there are more studies to explain this phenomenon.

A recent study reported that m6A-containing circRNAs could be degraded through YTHDF2–HRSP12–RNase P/MRP axis (Park et al., 2019). Mechanistically, human heat-responsive protein 12 (HRSP12) acted as an adaptor protein that linked YTHDC2 and RNase P/MRP to form YTHDF2-HRSP12-RNase P/MRP complex. Notably, after downregulating the expression YTHDF2, HRSP12, or POP1, Park et al. (2019) observed only three out of all tested 11 circRNAs commonly increased, indicating that this mechanism could only cleave a subset of m6A-containing circRNAs.

### m6A and Cellular Localization of circRNA

As we all know, a large number of circRNAs function in cytoplasm, such as miRNA sponges (Thomson and Dinger, 2016). Thus, it is essential to understand how the circRNAs export from the nucleus to the cytoplasm. Huang C. et al. (2018) found that *Drosophila* Hel25E and its human homologs, UAP56/URH49, could regulate circRNAs localization and control the efficiency of nuclear export by measuring the lengths of mature circRNAs. Chen et al. (2021) reported that YTHDC1 could bind to the m6A motif GAACU of circNSUN2 and facilitate circNSUN2 export from the nucleus to the cytoplasm, further promote colorectal liver metastasis by stabilizing HMGA2 (Chen R. X. et al., 2019). In addition, the m6A-modified circ1662 could bind to YAPI and accelerate its nuclear transport (Chen et al., 2021). However, more studies are required to investigate the details that m6A regulates the cellular localization of circRNAs.

## m6A circRNAS and Immunity

**FIGURE 2:**
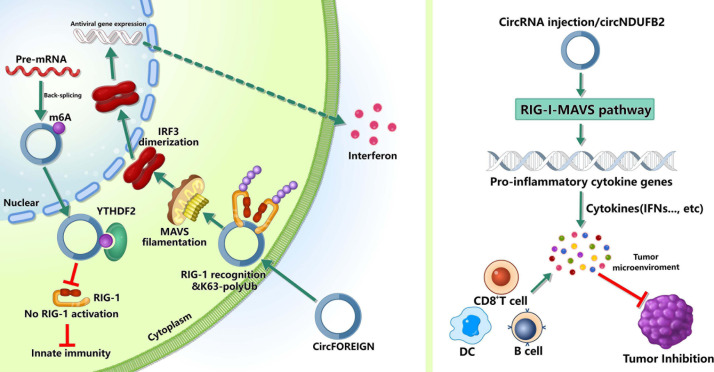
Roles of m6A-modified circRNAs in immunity. m6A can mark “self” circRNAs and these endogenous circRNAs inhibite innate immune responses, whereas exogenous circRNAs can activate innate immunity by activating RIG-I in the presence of K63-polyubiquitin. In tumor immunity, circRNAs may inhibit tumor progression by inducing anti-tumor immunity.

m6A in mRNA can control various aspects of immunity, including immune recognition, activation of innate and adaptive immune responses (Shulman and Stern-Ginossar, 2020). However, its roles in circRNA immunity are unknown. Several recent studies have discovered that m6A can also control circRNA immunity (Figure 2), including the roles in innate immune and anti-tumor immunity.

The innate immune system relies on pattern recognition receptors(PRRs) recognizing pathogen associated molecular patterns (PAMPs) to recognize “non-self” (Reikine et al., 2014; Wu and Chen, 2014). The PRRs, such as RIG-I and MDA5, can sense foreign nucleic acids. MDA5 can detect long double-stranded RNA (dsRNA) and RIG-I can recognize 5′ triphosphate on short dsRNAs (Schlee, 2013; Reikine et al., 2014; Wu and Chen, 2014). Reportedly, some exogenous circRNAs can activate antiviral and immune gene expression (Chen et al., 2017), while some endogenous circRNAs can inhibit protein kinase R and set the threshold for innate immunity upon virus infection (Liu et al., 2019). However, the mechanisms that the innate immune system recognizes self and non-self circRNAs remain unclear. Chen et al. (2021) found that m6A can mark circRNA as “self” and these endogenous circRNAs inhibited innate immune responses (Chen Y. G. et al., 2019). This immunosuppression was mediated by binding to YTHDF2 directly and inhibiting RIG-I activation. In addition, exogenous circRNAs activated innate immunity by directly binding RIG-I and K63-polyubiquitin chain and they together formed a three-component signaling-competent complex for immune signaling (Chen Y. G. et al., 2019). These evidence suggested that, for human circRNAs, m6A modification was essential for the recognition function of innate immunity.

Besides participating in the recognition of innate immunity, m6A-modified circRNAs can inhibit the tumor growth by inducing anti-tumor immunity. Chen et al. (2021) reported that circFOREIGN could induce CD8^+^ T cell responses, and after receiving circFOREIGN, the growth of tumor in mice model was lower than the negative control, indicating that circFOREIGN would can induce adaptive immunity against OVA-expressing tumors. Notably, Li et al. (2021) reported that the immune responses induced by m6A-modified circNDUFB2 could inhibit NSCLC progression. The overexpression of circNDUFB2 markedly inhibited the tumorigenicity of LLC1 cells (a murine lung carcinoma cell line) *in vivo*, and the level of IFN-β in serum was significantly increased in the mice with circNDUFB2-overexpressed LLC1 cells. Moreover, the infiltration of CD8^+^ T cells and DCs in the tumor tissues were significantly increased. These results suggested that m6A-modified circNDUFB2 inhibited tumor progression by activating anti-tumor immunity. However, more studies are needed to explore the roles of m6A modification in circRNAs in the field of tumor immunity.

These evidence demonstrated the roles of m6A modification in circRNAs during recognizing innate immunity and inducing anti-tumor immunity, but major knowledge gaps in field of immunity remain to be filled, such as its specific roles in different immune diseases and immune cells.

## m6A circRNA and Diseases

**FIGURE 3:**
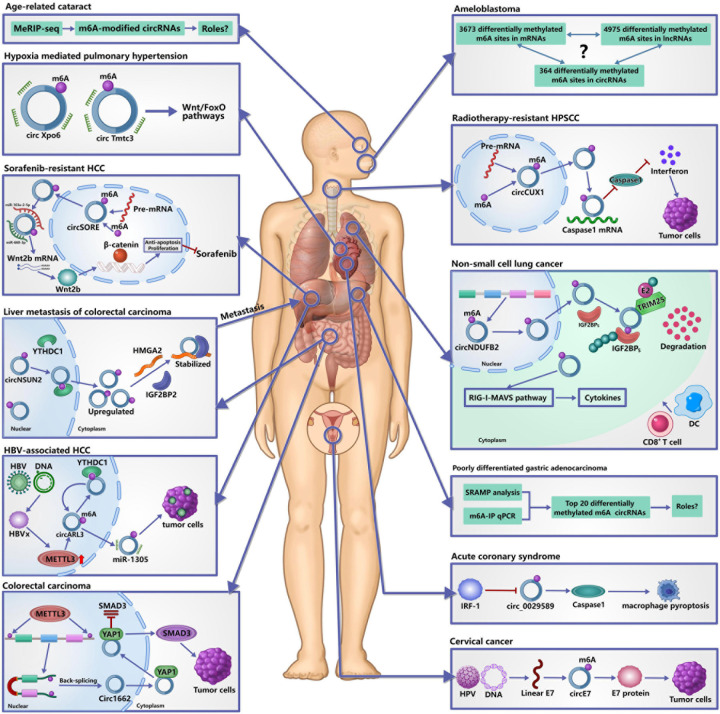
Roles of m6A-modified circRNAs in diseases. It has confirmed that m6A modification of circRNAs plays important roles in the occurrence and development of human diseases. In tumors, m6A-modified circRNAs are not only involved in their occurrence, development and metastasis, but also in the resistance of chemotherapy and radiotherapy by regulating the metabolism and functions of circRNAs. In acute coronary syndrome, IRF-1 facilitates macrophage pyroptosis and inflammation by inhibiting m6A-modified circ_0029589, thereby promoting the formation of necrotic core. In hypoxia mediated pulmonary hypertension, m6A may affect the interaction between circRNAs and miRNAs to participate in its development. In age-related cataract, many differentially expressed m6A-modified circRNAs are identified, but the specific roles remain unclear.

Although it was not until 2017 that circRNAs were found to be modified by m6A, many studies have shown that m6A modification of circRNAs plays important roles in physiological processes and disease progressions (Figure 3), such as tumors (Chen R. X. et al., 2019; Chen et al., 2021; Li et al., 2021), acute coronary syndrome (Guo et al., 2020), pulmonary hypertension (Su et al., 2020), and age-related cataract (Li et al., 2020). In this section, we focused on the roles of m6A-modified circRNAs in physiological processes and disease progressions.

### m6A circRNA and Tumor

m6A modification of circRNAs has been shown to be involved in theprogression of many tumors. In patients with colorectal carcinoma (CRC), metastasis is the overwhelming cause of death, and the liver is the most frequent distant metastatic site (Hofheinz and Stintzing, 2019). Recently, Chen R. X. et al. (2019) reported a novel metastatic mechanism to liver of CRC. First, YTHDC1 binds to the m6A motif of circNSUN2 and facilitates circNSUN2 export from the nucleus to the cytoplasm. Next, IGF2BP2 binds to the CAUCAU motif of circNSUN2 through the KH3-4 di-domain and the AAACA site inside of circNSUN2 can directly bind to the 3′UTR of HMGA2 with AU-Rich Elements, ultimately forming circNSUN2/IGF2BP2/HMGA2 RNA-protein ternary complex to stabilize HMGA2 mRNA, thereby promoting colorectal liver metastasis. This study provided the comprehensive evidence that m6A modification of circRNAs may contribute to cancer therapy (He et al., 2021). Another study found that m6A could increase the expression of circ1662 in CRC and circ1662 directly bound to yes-associated protein 1 (YAP1) and accelerated its nuclear accumulation to regulate the SMAD3 pathway, thereby promoting the invasion and migration of CRC cells (Chen et al., 2021).

In Hepatitis B virus (HBV)-associated hepatocellular carcinoma (HCC), circARL3 was upregulated in HBV positive HCC tissues and knockdown of circARL3 inhibited the proliferation and invasion of HBV + HCC cells (Rao et al., 2021). Rao et al. (2021) found that HBx protein could upregulate the expression of METTL3, increasing the m6A modification of circARL3. m6A-modified circARL3 can be read by YTHDC1 and regulate its biogenesis. Finally, circARL3 promoted HBV + HCC progression by sponging miR-1305.

In non-small cell lung cancer (NSCLC), circNDUFB2 was downregulated and elevated circNDUFB2 could inhibit growth and metastasis of NSCLC cells (Li et al., 2021). circNDUFB2 functions as a scaffold to enhance the interaction between TRIM25 and IGF2BPs and can from a TRIM25/circNDUFB2/IGF2BPs ternary complex. By the way, TRIM25 is an RNA-binding protein and belongs to the Tripartite Motif (TRIM) family of E3 ubiquitin ligases, which catalyzes the addition of polyubiquitin chains to its substrates for degradation (Lee et al., 2018). This ternary complex facilitates ubiquitination and degradation of IGF2BPs during NSCLC progression. m6A modification of circNDUFB2 can enhance this effect by regulating the interactions between circNDUFB2 and IGF2BPs. In addition, circNDUFB2 participates in the activation of anti-tumor immunity during NSCLC progression by activating the RIG-I–MAVS pathway (Li et al., 2021), indicating that circNDUFB2 inhibits NSCLC progression in a dual-role pattern.

In cervical cancer, Zhou et al. (2017) identified a large number of m6A-modified circRNAs in HeLa cells, indicating that m6A-modified circRNAs may play an important regulatory role in cervical cancer. As we all know, human papillomavirus (HPV) infection is the main cause that leads to the occurrence of cervical cancer (Chibwesha and Stringer, 2019). Zhao et al. (2019) reported that HPV could generate the circE7, which was an oncoprotein-encoding circRNA. circE7 is m6A-modified and can be translated to produce E7 oncoprotein in an m6A-independent manner. Specific disruption of circE7 can reduce E7 protein levels in CaSki cervical carcinoma cells and can inhibit cancer cell growth both *in vitro* and in tumor xenografts.

In poorly differentiated gastric adenocarcinoma (PDGA), Zhang et al. (2020) identified 20 differentiated expressed m6A-modified circRNAs by SRAMP analysis, m6A-immunoprecipitation and real-time quantitative PCR. They found that the trend of m6A modification alteration was mainly consistent with the expression level of circRNAs. However, the roles of these m6A-modified circRNAs in PDGA remain unclear and need to be explore extensively.

In human ameloblastoma, Niu et al. (2020) investigated the global m6A modification, including in mRNAs, lncRNAs and circRNAs. They found 3,673 differentially methylated m6A sites in mRNAs, 4,975 differentially methylated m6A sites in lncRNAs and 364 differentially methylated m6A sites within circRNAs. Although a global data of m6A in ameloblastoma was provided, their roles and the relationships among these m6A-modified mRNAs, lncRNAs and circRNAs need to be further explored.

Based on the above analysis, we can confirm that m6A modification of circRNAs plays critical roles in occurrence, development and metastasis of tumors. Of note, besides the above roles, m6A modification of circRNAs is also related to resistance of chemotherapy and radiotherapy. In sorafenib-resistant HCC, m6A could increase the expression of circRNA-SORE and increased circRNA-SORE sequestered miR-103a-2-5p and miR-660-3p by acting as a miRNA sponge, thereby competitively activating the Wnt/β-catenin pathway and inducing sorafenib resistance (Xu et al., 2020). In hypopharyngeal squamous cell carcinoma (HPSCC), m6A could stabilize the expression of circCUX1 and knocking down circCUX1 promoted the sensitivity of hypopharyngeal cancer cells to radiotherapy. Mechanistically, circCUX1 bound to caspase1 and inhibited its expression, leading to a decrease in the release of inflammatory factors, thereby developing tolerance to radiotherapy (Wu et al., 2021). As a key inflammation-related molecule, caspase1 can affect the occurrence, development, invasion, and metastasis of tumors by regulating the tumor inflammatory microenvironment (Hu et al., 2010), and caspase-1 actively induced tumor cell programmed death and anti-tumor immune surveillance (Zitvogel et al., 2012). Therefore, these evidence provided the new insights into understanding the resistant to radiotherapy.

### m6A circRNA and Acute Coronary Syndrome

Reportedly, macrophage death plays a key role in promoting the formation of necrotic core and plaque disruption in late atherosclerotic lesions (Hizir et al., 2017; Xu et al., 2018; Guo et al., 2019). Pyroptosis is a newly identified inflammatory programmed cell death with the activation of caspase-1 and the consequent release of inflammatory cytokines (Liu et al., 2016), and has been found to have a strong correlation with ox-LDL-induced human macrophage death and aggravates the instability of atherosclerotic lesions (Yan et al., 2016; Martinet et al., 2019). Guo et al. (2019) found that IFN regulatory factor (IRF)-1 could activate ox-LDL-induced macrophage pyroptosis in atherosclerosis (AS) and acute coronary syndrome (ACS). However, the underlying mechanism is unknown. A recent study found that the expression of hsa_circ_0029589 was significantly decreased in ACS and the relative m6A level of circ_0029589 was obviously elevated (Guo et al., 2020). Inhibition of IRF-1 obviously induced the expression of circ_0029589 and reduced the m6A level of circ_0029589, and knocking down the expression of METTL3 markedly increased circ_0029589 expression and decreased the m6A level of circ_0029589 and caspase-1 activity in AS and ACS (Guo et al., 2020). These results suggested that IRF-1 facilitated macrophage pyroptosis and inflammation in ACS by inhibiting circ_0029589 through promoting its m6A modification.

### m6A circRNA and Pulmonary Hypertension

Pulmonary hypertension is a lethal disease and hypoxia mediated pulmonary hypertension (HPH) belongs to group III pulmonary hypertension, which is induced by chronic hypoxia (Pugliese et al., 2015; Galiè et al., 2016). To investigate the significance of m6A circRNAs in HPH, Su et al. (2020) analyzed transcriptome-wide map of m6A circRNAs in a rat model by high-throughput m6A and circRNAs sequencing. They found that m6A abundance in circRNAs was significantly reduced under hypoxia *in vitro* and m6A-modified circXpo6 and circTmtc3 might be involved in HPH through regulating Wnt and FoxO signaling pathways by influencing the interactions between circRNAs and miRNAs. However, there are more evidence to explore the roles of m6A-modified circXpo6 and circTmtc3 in HPH and their bindings to miRNAs need to be validated by dual-luciferase reporter assay.

### m6A circRNA and Age-Related Cataract

Age-related cataract (ARC) is one of the leading causes of vision impairment and accounts for the majority of senile blindness worldwide (Khairallah et al., 2015). However, the pathogenesis of ARC remains unclear. A recent study found that there are a lot of m6A circRNAs in the lens epithelium cells from the patients with ARC (Li et al., 2020). The level of m6A abundance in total circRNAs was decreased compared with the controls and ALKBH5 was significantly upregulated in ARCs. The authors predicted the potential functions of m6A modified circRNAs and found the relevant pathways that may be associated with m6A modified circRNAs. These results indicated that m6A modification of circRNAs may play an important role in ARC. However, a large number of studies are needed to investigate their roles and validate the predicted the potential functions of m6A modified circRNAs.

Based on the above analysis regarding the roles of m6A modified circRNAs in different diseases, we can deduce that m6A modification in circRNAs has following common mechanisms to participate in disease progression. (1) Under pathological conditions, the expression of methyltransferases and/or demethylases is upregulated or downregulated, leading to the changes in m6A levels of circRNAs. (2) Through recognizing m6A motif by different m6A readers, m6A modification can regulate the metabolism and functions of circRNAs, thereby participating in disease progression (Figures 2, 3). However, the relationships among m6A modification, circRNA functions and diseases remain largely unclear. It is confirmed that m6A modification is as the upstream regulatory mechanism to affect downstream molecules by regulating circRNAs directly. In the future, the conjoint analysis of MeRIP-seq and RNA-seq may be helpful for seeking disease-specific m6A modified circRNAs. After that, qRT-PCR, RIP, RNA pull down, and dual-luciferase reporter assay may be used for exploring the mechanism of selected m6A modified circRNAs.

## Conclusion and Future Directions

No doubt, increasing number of studies have validated that circRNAs play key roles in human development and disease progressions; and epigenetic modification, especially m6A in mRNA, has emerged as widespread regulatory mechanisms that control gene expression in diverse physiological and pathological processes. However, the roles of epigenetic modifications in circRNAs remain largely unknown. Despite there are only 4 years regarding the researches of m6A in circRNAs, accumulating studies have identified numerous m6A-modified circRNAs and have found their important regulatory roles in development and diseases. In conclusion, current studies have found that these m6A-modified circRNAs are expressed in cell-type and disease-specific methylation patterns. In functions, m6A modification can regulate circRNAs metabolism, including circRNAs biogenesis, translation, degradation and cellular localization. Importantly, these m6A-modified circRNAs participate in diverse physiological and pathological processes, such as immunity, tumors, acute coronary syndrome, pulmonary hypertension and age-related cataract, by regulating circRNAs metabolism and functions. In general, the discovery of m6A modification in circRNAs broadened our horizons in RNA epigenetics and we provided a comprehensive understanding about the m6A modification of circRNAs and their roles based on current reports.

In addition, m6A-modified circRNAs may become the potential therapeutic target of diseases. For example, m6A-modified circNSUN2 has been found to play a key role in promoting colorectal liver metastasis (Chen R. X. et al., 2019; He et al., 2021). Targeted inhibition of circNSUN2 may block colorectal liver metastasis. Here, we listed the prospect targets that may be used for clinical treatment in the future in Table 1 based on current reports. However, the research regarding targeting m6A-modified circRNAs in diseases is still in infancy and many studies are needed to seek disease-specific targets and to develop effective methods for detection of m6A-modified circRNAs.

**TABLE 1:** m6A-modified circRNAs as the potential therapeutic target of different diseases based on current reports.

**Target**	**Disease types**	**Expression**	**Function**	**References**
circNSUN2	CRC	Up	Stabilize HMGA2 mRNA, and promote colorectal liver metastasis.	Chen R. X. et al., 2019
circ1662	CRC	Up	Bind to YAP1 and accelerate its nuclear accumulation to regulate the SMAD3 pathway.	Chen et al., 2021
circARL3	HBV + HCC	Up	Sponge miR-1305 and promote HBV + HCC progression.	Rao et al., 2021
circNDUFB2	NSCLC	Down	(1) Function as a scaffold to enhance the interaction between TRIM25 and IGF2BPs, from a TRIM25/circNDUFB2/IGF2BPs ternary complex and facilitate ubiquitination and degradation of IGF2BPs. (2) Participate in the activation of anti-tumor immunity by activating the RIG-I–MAVS pathway	Li et al., 2021
circE7	Cervical cancer	Up	Can be translated to produce E7 oncoprotein and promote the growth of cervical cancer.	Zhao et al., 2019
circRNA-SORE	Sorafenib-resistant HCC	Up	Sponge miR-103a-2-5p and miR-660-3p, thereby competitively activating the Wnt/β-catenin pathway and inducing sorafenib resistance.	Xu et al., 2020
circCUX1	Radiotherapy-resistant HPSCC	Up	Bind to caspase1 and inhibit its expression, leading to a decrease in the release of inflammatory factors.	Wu et al., 2021
circ_0029589	ACS	Down	Decrease caspase-1 activity.	Guo et al., 2020
circXpo6	HPH	Up	Sponge miRNA.	Su et al., 2020
circTmtc3	HPH	Up	Sponge miRNA.	Su et al., 2020

*CRC, colorectal carcinoma; HCC, hepatocellular carcinoma; HBV, Hepatitis B virus; NSCLC, non-small cell lung cancer; HPSCC, hypopharyngeal squamous cell carcinoma; ACS, acute coronary syndrome; HPH, hypoxia mediated pulmonary hypertension; YAP1, yes-associated protein 1; HMGA2, high mobility group AT-hook 2; TRIM25, Tripartite Motif 25; SMAD3, SMAD family member 3; RIG-I, retinoic acidinducible gene-I; MAVS, mitochondrial anti-viral signaling protein; IGF2BPs, insulin-like growth factor 2 mRNA-binding proteins.*

Currently, many questions are about when and how m6A is added on or removed from circRNAs, and about how m6A regulates circRNAs metabolism and disease progressions. For example, Tang et al. (2020) found that, during murine spermatogenesis, for a subset of circRNAs, the back splicing occurs mostly at m6A enriched sites, which are usually located around the start and stop codons in linear mRNAs. Zhou et al. (2017) found that numerous m6A circRNAs were generated from exons that didn’t contain m6A peaks in corresponding mRNAs. The question is whether m6A was added before or after the circRNAs were formed. In addition, the causes that lead to the change of m6A modification in circRNAs are unclear.

Another challenge is the technologies to study m6A modification. For example, MeRIP-Seq is the main method to identify m6A modifications using m6A-specific antibodies. A limitation of this method is that it cannot provide the precise location of m6A at single-nucleotide resolution. Ho-Xuan et al. (2020) found that endogenous proteins could not be detected when they constructed the overexpression of circZNF609. Comprehensive mutational analysis found that deletion constructs, which were deficient in producing circZNF609, still generated the observed protein products, suggesting that the apparent circZNF609 translation originated from transsplicing byproducts of the overexpression plasmids. Therefore, the overexpression construction of circRNAs need to be evaluated carefully. In the future, studies should overcome and solve these challenges and questions in order to better understand the roles of m6A in circRNAs biology further.

## Author Contributions

HS and LT designed and executed the study. JW and XG collected and analyzed the data, and wrote the manuscript. YW and SH made the figures. XY was responsible for language quality. All authors contributed to the article and approved the submitted version.

## Conflict of Interest

The authors declare that the research was conducted in the absence of any commercial or financial relationships that could be construed as a potential conflict of interest.

## Publisher’s Note

All claims expressed in this article are solely those of the authors and do not necessarily represent those of their affiliated organizations, or those of the publisher, the editors and the reviewers. Any product that may be evaluated in this article, or claim that may be made by its manufacturer, is not guaranteed or endorsed by the publisher.

## Footnotes

1 https://www.biosino.org/transcirc/
